# An assessment of nursing mothers’ and young people’s access to proprietary and patent medicine vendors’ services in rural communities of south-eastern Nigeria: implication for review of national drug policy

**DOI:** 10.1186/s40545-021-00334-7

**Published:** 2021-11-16

**Authors:** C. J. Uneke, I. Obeka, B. I. Uneke, A. Umeokonkwo, C. A. Nweze, K. I. Otubo, O. E. Uguru

**Affiliations:** African Institute for Health Policy & Health Systems, Ebonyi State University, PMB 053, Abakaliki, Nigeria

## Abstract

**Background:**

Patent and proprietary medicine vendors (PPMVs) form part of the informal healthcare system and are the first point of call for 75% of Nigerians who live in rural and underserved areas where there is limited access to healthcare services. This group of healthcare providers are located close to communities and are easily accessible to the people. This study seeks to determine how PPMVs influence access to medicines among nursing mothers and young people and how this progresses South Eastern Nigeria towards universal health coverage.

**Methods:**

A cross-sectional descriptive study was conducted using a purposive sampling technique. Two slightly different pre-tested and validated 5-point Likert scale questionnaires were used to survey the nursing mothers and young people (18–20 years old). The questionnaire for nursing mothers assessed the perception regarding PPMV services and community access to medicines used for the treatment of childhood infections. The questionnaire for young people assessed the services rendered by the PPMVs including family planning, and major enablers/barriers towards to access to medicine.

**Results:**

A total of 159 nursing mothers and 148 youths participated in the study. Up to 60% of both population had a minimum of secondary school qualification. About 90% of the nursing mothers were married and 88% were nursing babies from 1 to 12 months. Results show that the PPMVs were the first point of call for healthcare needs among the respondents and they are easily accessible and sell affordable medicines. The nursing mothers frequently treat their children’s cough with antibiotics with a mean rating (MNR) of 4.7 out of 5 points and most source these antibiotics from PPMV shops. Up to 90% of the nursing mothers reported that the children got well after the treatment. The drugs mostly purchased by the youths from the PPMVs included antimalarials (95%), analgesics (87.7%) and antibiotics (81.3%). Only 25.5% of the respondents purchased family planning commodities. Most of the respondents sought health care from PPMVs with a MNR of 3.4. Patronage of PPMVs for and usage of family planning products by the respondents had MNRs ranging from 1.4 to 1.8.

**Conclusion:**

PPMVs are bridging the gap in healthcare delivery in the rural and underserved areas. Training of this group of practitioners and appropriate monitoring will go a long way in ensuring that the services they render are efficient, effective and improve the health indices in a low-income setting.

## Introduction

Within the last decade, child health outcomes in Nigeria have somewhat improved, but the current situation is still unacceptably poor, especially in rural areas where health services are inadequate or completely lacking. With a population of more than 200 million, Nigeria has about 2.5% of the population of the world and 10% of all under-five deaths, translating into more than 1 million newborn, infant, and child deaths annually [1]. Maternal mortality ratio is estimated at 512 maternal deaths per 100,000 live births, and pregnancy-related mortality ratio is estimated at 556 pregnancy-related deaths per 100,000 live births [1]. Under-5 mortality rate is estimated at 132 deaths per 1000 live births, while infant mortality rate is estimated at 67 deaths per 1000 live births [1, 2]. This implies that more than 1 in 8 children in Nigeria die before their 5th birthday. Adult mortality rate is 3.18 deaths per 1000 population among women, and lifetime risk of maternal death indicates that one in 34 women in Nigeria will have a death related to maternal causes [1, 3]. Available reports indicate that Nigeria loses 2300 under-5 year olds every day, making the country the second largest contributor to under-five mortality in the world [3]. Another report showed that more than a quarter of a million neonates die in Nigeria each year, representing about 700 neonates per day [4]. It is important to state that most of these deaths are preventable, but the rural communities bear the greatest burden because of lack of timely access to medicines and healthcare.

According to the World Bank and United Nations Population Fund, of the estimated Nigeria population of 200,963,599 in 2019, about 22% (44.2 million) of the people are adolescents [5, 6]. The majority of this very high number of adolescents reside in rural areas. The lives of these young people are characterized by challenges in maintaining basic health and accessing health services, violence and abuse, dangerous behavioural activities due to peer pressure and difficult working conditions, all of which can negatively impact their health [7].

The Nigerian population is predominantly rural with over 75% of the population dwelling in rural areas where access to healthcare services provided by the formal sector are limited [8–10]. Consequently, the proprietary and patent medicine vendors (PPMVs), defined as individuals who have not received formal training in pharmacy but authorized to sell orthodox pharmaceutical products on a retail basis for profit [8, 9], are an important source of health care services. PPMVs shops are found in nearly all communities in Nigeria and are often the first and sometimes the only source of health care in those localities. They are the main access points for a range of health commodities and services, and are sought out for health advice and diagnosis, particularly amongst poor and rural populations with limited access to formal health services [10].

Available reports indicate that most young people in Nigeria, choose to patronize PPMVs over other services. The reasons are because the majority of young people cannot afford the very expensive private hospitals and clinics with professional staff and standardized services and also, they are unwilling to tolerate long waits, bureaucracies and costs at government-managed hospitals, which are currently understaffed and under-resourced [11].

An estimated 200,000 PPMVs operated in the country as of 2005, far outnumbering the 2639 retail pharmacies that were registered in the same year, and more than all other cadres of health workers in the country [12]. Unlike pharmacies which tend to be geographically concentrated in urban areas, PPMVs are more distributed across sub-urban and rural areas [13]. National surveys show that PPMVs are the first source of care for 55% of cases of fever, 30% of cases of diarrhoea, and 8% of cases of cough occurring amongst children under five [14]. Community- and state-level studies of care-seeking behaviour similarly find that PPMVs are the first source of care for up to 55% of under-five child illnesses, and provide services for 35% to 55% of adults seeking malaria treatment and remains the most preferred service by the poor [10, 15].

Due to the large numbers of PPMVs and their presence in rural areas, there are obviously regulatory challenges. However, policymakers and programme implementers in Nigeria are increasingly recognizing the need to engage PPMVs in a more efficient manner so they can contribute to the delivery of basic healthcare commodities and services to rural dwellers [10]. The Nigeria Drug Law Policy permits PPMVs to sell a limited number of pre-packaged, over-the-counter medicines and medical products to consumers, but prohibits the PPMVs from selling drugs categorized as prescription medications (e.g. antibiotics) and conduct medical procedure classified as invasive (e.g. injections) [16]. But it is in the recognition of the vital role of the PPMVs that is demonstrated by the fact that a number of Nigerian health policies and regulatory changes included them as important healthcare service providers especially in places where the formal sector cannot reach. For instance, following the revision of the national treatment guidelines for uncomplicated malaria with the recommendation of artemisinin-based combination therapy (ACTs) as the first-line treatment, the List of Essential Medicines was revised to authorize the PPMVs to sell ACTs [16].

The Nigerian Government has also approved the addition of paediatric zinc and co-packaged zinc and oral rehydration salts (ORS) to PPMVs’ list of approved medications as part of the national Essential Medicines Scale-Up Plan (EMSUP) [10]. Also included in the EMSUP is the implementation of continuous education and other forms of capacity development for PPMVs to improve their quality of care for common childhood illness [17]. PPMVs have also been listed as potential community-level implementers for the newly adopted national integrated Community Case Management (iCCM) guidelines [18], and have been included in a pilot project for home management of malaria under the National Malaria Strategic Plan [19]. The National Task Shifting and Sharing Policy issued in 2014 also calls for capacity building of community-oriented resource persons, including PPMVs, to provide treatment, counseling, and referral for some child health services [10].

In recent times there have been calls for more policy change to expand the scope of operations of the PPMVs to accommodate more services related to childhood illnesses and access to medicines by adolescents, but more evidence is needed to determine the safety and feasibility of this practice. In this study we assessed youths’ and nursing mothers’ access to PPMVs’ services, to provide scientific evidence that can guide possible policy change.

## Methods

### Study area

The study was conducted in Ebonyi State, south-eastern Nigeria. It is made up of 13 Local Government Areas (LGAs) divided into three senatorial-zones (North, Central, South), which are geopolitical zones designed to facilitate equity in resource allocation. The population of the State was put at 2,176,947 by the 2006 census. The 2018 population projected from 2006 census with a growth rate of 2.8% was 3,053,607 (48.9% males and 51.1% females). The average population density is 386 persons per square kilometre. Women of child bearing age (WCBA) (15–49 years) constitute 22% of the total population [9]. Besides very few semi-urban towns, all other areas are predominantly rural with about 75% of the population living in the rural and underserved communities and are predominantly farmers and traders.

According to the Ebonyi State Ministry of Health [9], major causes of infant and under-5 morbidity and mortality include: neonatal causes (26%), malaria (24%), pneumonia (20%), diarrhoea (16%), measles (6%), HIV/AIDS (5%) and others (3%). Most of these deaths occur in the rural areas where the formal healthcare services are lacking. Consequently, there is a large number of the informal providers in the private healthcare sector, and in particular owner-operated drug shops called patent and proprietary medicine vendors (PPMVs) operating in these rural communities. These PPMVs are the major sources of healthcare services in these rural communities of Ebonyi State. Three LGAs have been selected for the study with one from each senatorial zone: North (Izzi); Central (Ikwo); South (Ohaozara).

### Study design

The study design was a cross-sectional descriptive study technique. The participants included young people and nursing mothers.

### Survey of nursing mothers

A 5-point Likert scale structured questionnaire was developed and pre-tested in another LGA and validated. This enabled us to adjust the tool to optimize data collection. The development, pretesting, and validation of the questionnaire were done using the methods described by Tsang and colleagues [20]. First, a thorough consideration of issues relating to the format of the questionnaire and the meaning and appropriateness of the items were considered during the questionnaire development stage. Thereafter, a pilot test (undeclared pretest) was conducted on a limited number of respondents to ensure that the items were understood and correctly interpreted by the intended respondents. The questionnaire validation was done to ensure its reliability and that it was sound psychometrically. The questionnaire assessed the perception of the nursing mothers regarding PPMV services and community access to medicines, where they obtained them, what type of medicines they use for the treatment of childhood cough/acute respiratory infections, sources of advice/information on the treatment of coughs and colds and on the use of antibiotics, and type of advice the PPMVs give on treatment of coughs and cold for their children.

The inclusion criteria were as follows: (i) respondent must be a nursing mother; (ii) must been residing in the target LGAs for at least 6 months; and (iii) must have used the services of the PPMVs.

The nursing mothers were selected for this study because they are the care givers in their families and are usually the ones to seek medical attention for their children at the PPMVs shops. Nursing mothers attending immunization clinic at the government Primary Healthcare Centre (PHC) in each of the LGAs, were randomly recruited for the survey. Approval was obtained from the Executive Secretary of the Primary Healthcare Development Agency of the Ebonyi State and from the medical officer-in-charge of each of the target PHCs in the LGAs.

On each of the days of the survey, before the commencement of the immunization activities at the PHCs by the nurses, members of the research team were granted the opportunity to address the nursing mothers, introduced the study to them and invited them to participate. Consenting nursing mothers were administered with the questionnaire privately. Before giving any one the questionnaire, oral informed consent was obtained. We used part of the waiting time (maximum 20 min) of the mothers which is usually between 40 and 60 min or the time after the immunization, depending on what is most convenient for the respondent. All the mothers who could not speak English were interviewed and assisted to complete the questionnaire by members of the research team, who spoke the local language of the respondents. The research team also visited some additional nursing mothers, booked appointments with them for the survey, and at the appointed day and time at their respective homes, administered the questionnaire to them at the time most convenient to them.

### Survey of young people

We targeted up to 50 young people from each of the three LGAs. The study inclusion criteria included: (i) participant must be aged 18–20 years old; (ii) participant must be residing in the target LGAs for at least 6 months; (iii) participant must have used the services of the PPMVs in the locality.

Data collection was done using a structured pre-tested questionnaire similar to the one used for the nursing mothers but with some modification. The questionnaire was designed in a 5-point Likert scale which assessed PPMV services and access to medicines including family planning services and major enablers and barriers to access to services rendered by PPMVs. Other questions to youth explored commonest clinical presentations to PPMVs, commonly used medications, major sources of health advice/information, experience after accessing medicines from PPMVs, and cost of medicines from PPMVs.

A purposive sampling technique was used to identify consenting and willing participants. The researchers met participants in the streets and market places. The questionnaire was administered to each of the youths privately having assured each of them that information obtained was for research purposes and that confidentially of information given would be maintained. The questionnaire was administered by trained research assistants who were young and within the age bracket of the respondents to allow for easy interaction. The questionnaire was self-administered, but was interviewer administered if the subject was not literate. Written informed consent was obtained from each of the respondents and oral consent from the non-literate respondents. Maximum duration for the engagement per person was 20 min.

### Data analysis

Analysis of data collected via the 5-point Likert scale questionnaire was done using the methods developed at McMaster University Canada by Johnson and Lavis based on mean ratings (MNRs) [21]. For instance, the figures represent Likert rating scale of 1–5 points, where 1 point = grossly inadequate; 2 points = inadequate; 3 points = fairly adequate; and 5 points = very adequate. In terms of analysis, MNR ranging from 1.00 to 3.49 points were considered low, whereas MNR ranging from 3.50 to 5.00 points were considered high [22].

### Ethical approval

Ethical clearance was obtained from the University Research Ethics Committee (UREC) of Ebonyi State University Abakaliki (Ref: EBSU/DRIC/UREC/Vol. 05/047) and the Ethics Review Committee of the World Health Organization. Both the institutional and international guidelines on research ethics were strictly adhered to in all aspects of the project. Participant anonymity was maintained and all findings were treated with utmost confidentiality and for the purpose of the research only.

## Results

### Outcome of assessment of nursing mothers

A total of 159 nursing mothers (54 in Ohaozara LGA, 53 in Ikwo LGA, and 52 in Izzi LGA), participated in this study. Up to 62% of the nursing mothers were aged 21–30 years, had secondary education (60.56%), traders (46.3%), and had 1–2 children (41.1%). Most of the mothers (90.3%) were married and up to 69.7% of the respondents had lived in the community for upwards of 10 years. The age of the babies most of respondents were nursing was in the range of 1 month to 5 months (Table 1).

**Table 1:** Socio-demographic characteristics of the respondent nursing mothers in rural areas of Ebonyi State Nigeria

Characteristics	Frequency	Percent
Age (years)		
≤ 20	14	8.9
21–30	98	62
31–40	41	26
41–50	5	3.2
Total	158	
Marital status		
Single	14	9.0
Married	140	90.3
Widow/widower	1	0.6
Total	155	
Education		
No formal	2	1.3
Primary	38	24.5
Secondary	94	60.6
Tertiary	21	13.5
Total	155	
Occupation		
Student	2	1.6
Unemployed	16	13
Civil servant	18	14.6
Trader	57	46.3
Farmer	26	21.1
Hand craft	34	27.6
Total	123	
How many children you have		
1–2	65	41.1
3–4	45	28.5
5–6	33	20.9
≥ 7	15	9.5
Total	158	
How old is present child nursed		
1–5 months	90	57
6–12 months	49	31
13–18 months	14	8.9
≥ 19 months	5	3.2
Total	158	
Duration of residency in the community (years)		
1–3	18	11.8
4–6	19	12.5
7–9	9	5.9
≥ 10	106	69.7
Total	152	

The outcome of the nursing mothers’ perception of the PPMV services and community access to medicines is shown in Table 2. The MNRs for questions related to PPMVs being the first and often the only point of call for health care services; PPMVs being easily accessible; and PPMVs selling cheaper and more affordable drugs ranged from 3.2 to 3.6 on the scale of 5 points (Table 2). For the questions related to PPMVs sale of low quality, expired or sub-standard drugs; PPMVs not having the prerequisite training to operate shops, sale of medicines without prescription; and PPMVs having low health knowledge about proper treatment for common illnesses, the recorded MNR ranged from 3.3 to 3.8 (Table 2). In terms of the frequency of treatment of children’s coughs and colds with antibiotics in self-care (without consulting a health worker) by the mothers, the MNR recorded was 4.7. The MNR for their perceived efficacy of antibiotics and awareness of risks of antibiotics was 4.0 (Table 2).

**Table 2:** Nursing mothers’ perception of the PPMV services and community access to medicines in rural areas of Ebonyi State Nigeria

To what extent do you agree with the following (strongly disagree, disagree, unsure, agree, strongly agree)	Mean rating (out of 5-point Likert scale)
(i) PPMVs are the first and often the only point of call for health care services in this rural area	3.2
(ii) PPMVs are easily accessible to the rural dwellers	3.3
(iii) The medicines and drugs sold by the PPMVs are cheaper and more affordable than in hospitals and pharmacies	3.6
(iv) Many PPMVs in this locality sell low quality, expired or sub-standard drug to the rural dwellers	3.3
(v) Many of the PPMVs in this locality do not have the prerequisite training to operate PPMV shops	3.3
(vi) Sale of medicines without prescription is rampant in this locality	3.8
(vii) PPMV shop owners and operators generally have low health knowledge about proper treatment for common illnesses, such as cough, malaria and diarrhoea and poor health treatment practices	3.5
Frequency of treatment with antibiotics (never, rarely, occasionally, frequently, very frequently)	
How frequent do you treat your children’s coughs and colds with antibiotics in self-care (without consulting a health worker)	4.7
Effectiveness of treatment with antibiotics (don’t know, not effective, fairly effective, effective, very effective)	
How would you describe the effectiveness of these other treatments as compared to antibiotics?	3.3
Perceived efficacy of antibiotics and awareness of risks of antibiotics (grossly inadequate, inadequate, fairly adequate, adequate, very adequate)	
What is your perceived efficacy of antibiotics?	4.0
How adequate is your level of awareness of risks of antibiotics?	4.0

Up to 31.3% of the respondents credited their knowledge of drugs to PPMVs, while 57.1% of them accepted that the antibiotics they used in self-care were obtained from patent medicine stores or chemists (Table 3). Finding showed that 36.6% of the respondents commenced treatment on childhood acute respiratory infections (cough/cold) within 1 h of noticing the sickness, while 55% of the respondents noted that they administered antibiotics three times a day and 42% administered the antibiotics for 3 days. More than 90% of the respondents’ children got well as a result of administration of the antibiotics. Up to 65.5% of the mothers did not have to take their children to a health facility after treatment at home. Of the number that took their children to hospital 68.6% did so to ensure that the child is cured. Up to 46.4% of the respondents who agreed that they used non-drug therapy used herbs/herbal mixtures for treatment of their children (Table 3).

**Table 3:** Nursing mothers’ response on access to services provided by PPMVs for childhood illness treatment

Parameter assessed	Frequency	Percent
Sources of advice/information on the treatment of coughs and colds and on the use of antibiotics		
(a) Chemist/patent medicine vendor	52	31.3
(b) Health worker	61	36.7
(c) Neighbour	3	1.8
(d) Family members/relatives	44	26.5
Others	6	3.6
Total	166	
What kind of advice do the PPMVs give on treatment of coughs and cold for your child?		
Administer drugs including antibiotics	38	37.3
Cover the child with cloth	29	28.4
Use warm water	19	18.6
Go/refer to hospital	9	8.8
Come back if the sickness persists	7	6.9
Total	102	
Sources where antibiotics used to treat childhood cough/acute respiratory infections are obtained		
(a) PPMVs store	89	57.1
(b) Primary Health Centre	38	24.4
(c) General hospital	19	12.2
(d) Drug hawkers	1	0.6
(e) Others	9	5.8
Total	156	
The last time child had childhood acute respiratory infections, how many hours after noticing did you commence the treatment		
(a) Within 1 h	56	36.6
(b) Within 2 to 6 h	25	47.2
(c) Within 6 to 12 h	14	3.1
(d) Within 12 h to 1 day	29	19
(e) Within 1 day to 2 days	29	19
Total	153	
How many times in a day did you administer the drug?		
(a) Once	3	2
(b) Twice	61	40.4
(c) 3 times	83	55
(d) 4 times	1	0.7
(e) 5 times	3	2
Total	151	
For how many days did you give the drug?		
(a) 1 day	1	0.6
(b) 2 days	10	6.6
(c) 3 days	63	42
(d) 4 days	18	12
(e) 5 days	58	38.6
Total	150	
What was the outcome of the treatment you gave your child?		
(a) Got well	138	90.8
(b) Got a little better	14	9.2
Total	152	
Did you have to take your child to a health facility, dispensary or chemist after treatment at home with the drugs?		
Yes	51	34.5
No	97	65.5
Total	149	
If Yes, why?		
(a) Sickness persisted	15	29.4
(b) To be sure child is cured	35	68.6
(c) Sickness getting worse	1	2.0
Total	51	
What other treatments do you use (including non-drug therapies) to treat childhood cough/acute respiratory infections?		
Herbs/herbal mixture	58	46.4
Warm water	51	40.8
Balm	16	12.8
Total	125	

### Outcome of assessment of youths

A total of 148 youths participated in the study (53 in Ohaozara LGA, 46 in Ikwo LGA, and 49 in Izzi LGA). More than a third (44%) of the respondents were in their 20 years of age. 52.4% of the respondents were female and 60% had a secondary education. Fifteen percent of the respondents were married. Seventy percent of the respondents had lived more than 10 years in the study areas. More than half of the respondents lived with their parents (Table 4).

**Table 4:** Socio-demographic characteristics of the respondent youths in rural areas of Ebonyi State Nigeria

Characteristics	Frequency	Percent
Age (years)		
18	44	30.4
19	37	25.5
20	64	44.1
Total	145	
Sex		
Male	70	47.6
Female	77	52.4
Total	147	
Education		
No formal	1	0.7
Primary	21	15.2
Secondary	83	60.1
Tertiary	33	23.9
Total	138	
Marital status		
Single	122	84.1
Married	22	15.2
Separated	1	0.7
Total	145	
Occupation		
Student	78	53.8
Hand craft	36	24.8
Trader	11	7.6
Unemployed	10	6.9
Civil servant	4	2.8
Farmer	6	4.1
Total	145	
Duration of residency in the community (years)		
1–3	25	17.2
4–6	10	6.9
7–9	8	5.5
≥ 10	102	70.3
Total	145	
Home status		
Live alone	19	12.9
Live with parents	97	66.0
Living with guardians/relatives	12	8.2
Live with spouse/co-habiting	19	13
Total	147	

In terms of how often the respondents go to various sources of treatment for illness, the source with the highest MNR was PPMVs (3.4), followed by hospital (2.5) and health centre (2.4). The PPMVs being easily accessible to rural communities had MNR of 4.2 followed by discouraging dispensing medicine without prescription which had MNR of 4.0. Medicine availability without restriction being a big problem recorded MNR of 3.9, while MNR of 3.7 was recorded for PPMVs being the first and often the only point of care in rural area and sale of cheaper and affordable (Table 5). Patronage of PPMVs and usage of family planning products and sexually transmitted disease medications by the respondents had MNRs ranging from 1.4 to 1.8. Access to abortion or post-abortion care from the PPMVs by the female respondents had MNR of 1.4 (Table 5).

**Table 5:** Perception of youths on the PPMV services and young people’s access to medicines including family planning services

How often do you go to the following as sources of treatment for illness? (never, rarely, occasionally, often, very often)	Mean rating (out of 5-point Likert scale)
PPMVs	3.4
Hospitals	2.5
Health centres	2.4
Herbalists	1.8
Spiritualists	1.6
To what extent do you agree with the following (strongly disagree, disagree, unsure, agree, strongly agree)	
(i) PPMVs are the first and often the only point of call for health care services in this rural area	3.7
(ii) PPMVs are easily accessible to the rural dwellers	4.2
(iii) The medicines and drugs sold by the PPMVs are cheaper and more affordable than in hospitals and pharmacies	3.7
(iv) Many PPMVs in this locality sell low quality, expired or sub-standard drug to the rural dwellers	3.2
(v) Many of the PPMVs in this locality do not have the prerequisite training to operate PPMV shops	3.3
(vi) Sale of medicines without prescription is rampant in this locality	3.8
(vii) Dispensing medicines without prescription should not be encouraged	4.0
(viii) Medicines/drugs availability without restriction and control to public is big problem	3.9
(ix) PPMV shop owners and operators generally have low health knowledge about proper treatment for common illnesses, such as malaria and diarrhoea and poor health treatment practices	3.1
How often do you carry out the following? (never, rarely, occasionally, frequently, very frequently)	
(i) How often do you buy and use family planning products like condoms from PPMVs	1.7
(ii) How often do you buy and use family planning products like oral contraceptives from PPMVs	1.4
(iii) How often do you buy and use family planning products like contraceptives injections	1.4
(iv) How often do you buy and use sexually transmitted infections drugs/medicines from PPMVs	1.8
(v) How often have you being referred to hospitals by PPMVs for family planning related/reproductive health problems to hospitals?	1.4
(vi) How often have you being educated or advised by PPMVs on the options and usage of family planning products?	1.8
(vii) How often do access abortion/post-abortion care services from PPMVs? (*For females only*)	1.4

The drugs mostly purchased by the youths from the PPMVs included antimalarials (95%), analgesics (87.7%), antibiotics (81.3%), antiseptics (72.4%), haematinics (66.9%), and oral rehydration solution (62.8%). Only 37 (25.5%) of the respondents purchased family planning products (Table 6). Geographical proximity, low cost of medicine and good response (i.e. symptoms resolved) to care given were the three top enablers to respondents’ patronage of PPMVs. Major barriers to patronage of PPMVs were inadequate training of the PPMVs, sale of substandard/expired drugs and low educational qualification of the PPMVs (Table 6).

**Table 6:** Proportion of respondent youths that purchased various drug types from PPMVs and barriers/enablers to the use of PPMVs services

Variable	Frequency (*n* = 146)	Percentage (%)
Drug group name		
Antimalarial	134	95.0
Analgesics	128	87.7
Antibiotics	117	81.3
Antiseptics	115	72.4
Haematinics	95	66.9
Oral rehydration solution	91	62.8
Herbal mixture	80	54.8
Antitussives	79	55.6
Anthelminthic	77	54.6
Medical consumables	71	49.3
Zinc tablets	69	47.6
Antacids	64	44.8
Intravenous fluids	60	41.4
Parenteral drugs	55	38.5
Antihistamines	55	38.2
Family planning commodities	37	25.5
Anxiolytics	26	18.6
Antipsychotics	18	12.4
Antihypertensive	17	12.0
Antidiabetics	11	7.6
Enablers and barriers to the use of PPMVs by the respondents		
Enablers		
Closeness to the people	87	65.2
Cheap drugs	66	44.2
Good response to care	16	11.0
Good rapport with the people	13	8.9
No delay in accessing care	10	6.8
Access to credit facility	5	3.4
Barriers		
Sale of substandard/expired drugs	52	35.6
Lack of training	44	30.1
Low educational qualification	28	19.2
Fear of side effects	11	7.5
Lack of supervision	9	6.2
Lack of experience	6	4.1

The common illnesses for which the respondents visited the PPMVs for treatment were malaria, headache, and stomach ache (Table 7). The two most common sources of advice or information about medicine were from parents (42.2%) and from PPMVs (38.4%). The commonest used medicines were antimalarial (41.1%), paracetamol (38.4%), analgesics (36.3%), antibiotics (34.2%) and contraceptives (15.8%). A higher percentage (67.8%) of the respondents stated their symptoms improved after accessing care from PPMVs (Table 7).

**Table 7:** Summary of response from the youths on issues concerning access to medicines and health care services by PPMVs

Parameter assessed	Frequency (*n* = 146)	Percent (%)
Common illness		
Malaria	117	71.3
Headaches	76	51.2
Stomach ache	45	30.8
Cough/cold	36	24.7
Typhoid	32	21.9
Body pain/weakness	29	19.9
Fever	16	11.0
Source of advice/information on medicines		
Parents	68	42.2
PPMVs	56	38.4
Friends	33	22.6
Other health workers	30	20.5
Doctors	18	12.3
Relatives	11	7.5
Media	10	6.8
Commonly used medicines		
Antimalarial	60	41.1
Paracetamol	56	38.4
Analgesics	53	36.3
Antibiotics	50	34.2
Contraceptives	23	15.8
Tramadol	22	15.1
Vitamins	18	12.3
Outcome after accessing care from PPMVs		
Got well	99	67.8
Got a little better	38	26.0
No improvement	5	3.4
Got worse	4	2.7

## Discussion

The outcome of this study suggests that the PPMVs are the first and often the only point of call for healthcare services among nursing mothers in the rural areas and that they are easily accessed by those that live in rural areas. In Nigeria, owner-operated drug retail outlets or PPMVs are the main source of medicine for acute conditions [19]. In particular, drug shops comprise a sizeable portion (nearly 40%) of the private healthcare sector in Nigeria and provide between 80 and 90% of all child health services in rural areas [10, 11]. Durowade and associates [23] in their study showed that majority of the clients could access PPMV shops within 2–30 min’ walk and that clients could go to the PPMVs houses or call them on phone after closing hours. This study further corroborated the findings in the study conducted by Iheoma and co-workers [24] where they noted that PPMVs are accessible, affordable and are valuable sources of healthcare services, products and information.

The results also clearly suggest that the PPMVs remain the most popular source of medicine and health services among young people. The reasons for their choice of the PPMVs include geographical proximity, cheap drugs, good response to care (i.e. symptoms resolved), no delay in accessing care, good rapport with the people and access to credit facility are consistent with previous studies from Nigeria [7, 11, 25]. In addition to these enablers, Okonkwo and Okonkwo [11] added that Nigerian youths prefer PPMVs because they are more accessible to young patrons than public sector health service providers; display non-judgemental attitudes towards young people’s sexual healthcare needs; they are diplomatic, confidential and offer a wide array of sexual health services to youth, despite policy regulations that constrain this.

PPMVs have played vital roles in the health care system in different parts of Nigeria. In assessing the enablers and barriers to the use of PPMVs, we found that the geographical proximity of PPMVs was a major enabler to their patronage by the young people in the study area. This finding has been corroborated by earlier studies which reported that PPMVs located in an area close to the community was a strength for their operation [11, 26, 27]. This has also been noted by PPMVs themselves as reported by Sieverding and Beyeler [26]. This finding is important as it reflects that geographical barriers present a challenge to accessing healthcare, which is critical to address when progressing towards achieving universal health coverage. In most rural communities where there are access challenges, PPMVs fill the gaps. They are perceived as the frontline health workers in these communities, playing an important primary health care function [28]. Since the PPMVs already have some trust and rapport with the community and can provide some basic services, they can play an important role in healthcare delivery and should be considered as an integral part of Nigeria’s strategy towards universal health care. They could be made more functional through proper training and regulation.

Majority of the young people in this study, affirmed that drugs sold by PPMVs were cheaper and more affordable than those in hospitals and pharmacies. This is in agreement with the study by Iheoma and co-workers [24] they acceded to the fact that drugs purchased from PPMVs are cheaper. A significant proportion of the respondents reported that the affordable cost of products and services encouraged their use of services provided by PPMVs. Similar observations have been reported earlier [27]. Financial barriers to accessing the formal health system have also been reported as reason for patronage of PPMVs, as they cannot afford services of private hospitals and clinics [19]. In this study, other enablers found, regarding the patronage of PPMVs by young people, just as the nursing mothers, were their good response to given care (resolution of symptoms) by the PPMVs, short waiting time and friendly nature of PPMVs.

The resolution of symptoms, short waiting time and friendly nature of PPMVs were reported to encourage patronage of PPMVs in previous studies [29–31]. These findings draw attention to some gaps to in the formal health system that need urgent attention. The importance of patient satisfaction to encourage accessing health services and the negative effects of prolonged waiting time on service uptake have been reported [32–34]. Similarly, the absence of health insurance coverage and the subsequent financial impact of out-of-pocket spending should be considered for their implications in achieving universal health coverage (UHC). In a bid to avoid catastrophic health spending that may result from accessing health care in the formal setting, these young people are attracted to the cheaper alternatives provided by the PPMVs.

This study showed that the nursing mothers agreed that PPMVs sell low quality medicines. This is in consonance with earlier studies which showed that nearly half of antimalarial drugs stocked in PPMV shops were sub-standard or expired [35, 36]. PPMVs generally have low health knowledge and poor treatment practices, stock poor quality medicines (e.g. partial or repackaged doses) [37] and substandard formulations [38] as well as commodities they are prohibited to sell [39]. Sale of substandard and expired drugs by the PPMVs was also one of the barriers reported by the young people in our study. This may be related to weak enforcement of the set down guidelines and regulations by the regulatory authority [34]. The implication of this finding is that if the PPMVs are not strictly regulated and supervised, they may likely contribute to increased morbidities and mortalities attributable to use of substandard and expired drugs.

A fairly significant population of the nursing mothers felt that PPMVs in their locality do not have the prerequisite training to operate PPMV shops. They also agreed that operators generally have low health knowledge about proper treatment for common illnesses. This is so because conventionally, the minimum educational attainment stipulated by PCN for registration of PPMVs has been primary schooling [24] and Egbohin his work [40] stated that formal medical or pharmacy training is not required for PPMV licensure. Our study showed that perceived lack of training of PPMV staff was one of the main barriers to seeking health care from them among youth. Despite this, youth still accessed their services, potentially due to limited availability of alternatives or financial barriers to accessing the formal health sector. This perceived lack of training of PPMV staff is supported by Oyeyemi et al. [41] who reported that only a third of PPMVs had previous health related training. Additionally, Chiaka and colleagues [29], showed that PPMVs are not knowledgeable in diagnosis and treatment of diseases.

A systematic survey carried out by Beyeler and associates [25] noted that most PPMVs have low health knowledge and poor health treatment practices. For the PPMVs to give appropriate and timely advice to caregivers who tend to the sick children, it is necessary that they have basic knowledge of the presentation, cause, treatment and prevention of childhood diarrhoea [43]. However, Liu and his workers noted that majority of shop owners they assessed in their study in parts of Nigeria, have completed secondary or post-secondary education [10]. With a significant association demonstrated between knowledge and education level, an opportunity is presented for policies and interventions that emphasize education and training of PPMVs to improve their knowledge on childhood diarrhoea (and perhaps other childhood illnesses?) and to function optimally in primary care delivery in the community [42, 43].

The respondents fully agreed that sale of medicines without prescription in their locality was rampant. This study validates the result of the study by Akinyandenu and colleagues [43] where they noted that the sale of antibiotics without medical prescription has been observed in many countries [39]. A study conducted in Nairobi showed that about 64% of chemists sold antibiotics without prescription [36]. Antimalarials, analgesics, antibiotics and antiseptics were the most purchased drugs from PPMVs in our study. This outcome is related to the commonly endemic illness and injuries in rural setting and has been corroborated in earlier studies [27]. It is also instructive to note that some prescription-only medications are sold by these PPMVs. This clearly points to weak regulation of these drugs.

Most nursing mothers in this study agreed that they treat their children’s cough and cold with antibiotics in self-care without consulting a health worker. This is in tandem with the study which noted that 80% of illness episodes are self-treated with medicines obtained from community pharmacies [44]. This study further revealed that more than half of the respondents obtained these antibiotics used in the treatment of childhood cough and cold from PPMVs. Indalo [45] in his study showed that most chemist shops in peri-urban areas sold antibiotics without prescription.

A considerable proportion of the nursing mothers, noted that they sought for treatment of their children’s cough within 1 h of noticing the sickness. This must have been made possible because the respondents are nursing mothers, who given their close contact with children were frequently the first to identify illness symptoms [46]. Most of these mothers had a minimum of secondary schooling qualification. This agrees with a study conducted in Cross River and Bauchi States of Nigeria where they noted that mothers who have not attended school have lower rates of appropriate care seeking when compared to those that have attended school [47]. Appropriate care seeking involves identifying the need to take the child for treatment outside the home, ensuring that the care is not delayed and that the child is taken to appropriate health facility or provider [48].

Drug advice on the treatment of coughs and colds and on antibiotic use were obtained from patent medicine vendors and from health workers. This work agrees with the study by Prach and co-workers [36] where they noted that in addition to selling drugs patent medicine vendors can be a source of advice about illness and drug therapy [49]). The work done by Abdu-Aguye [50] showed that patent medicine vendors knowledge about antibiotics is moderate and needs to be enhanced by further training to ensure that they do not transfer wrong knowledge to their clients [50].

The respondents self-reported perceived efficacy of antibiotics was very high, with up to 90.8% reporting that their children got well after antibiotic treatment. The awareness of antibiotics could have been made possible by the information provided to them during counselling by PPMVs and health workers. This gives credence to the findings of Ibeneme and colleagues [29] where they noted that fewer than half of the mothers who resorted to PPMVs for treatment of childhood febrile conditions perceived that their child had fully recovered. However, in this study a few of the mothers noted that the sickness persisted after treatment. Okeke and his colleagues [39] noted that this persistence could be due to misdiagnosis and/or mismanagement of the health condition.

Interestingly, among the young people, our finding showed that only 25.5% of the respondents have ever purchased family planning products. Furthermore, the MNR for the questions related to the frequency of buying and using family planning products like condoms, oral contraceptives and contraceptives injections was very low, ranging from 1.4 to 1.7 out of 5-point Likert scale. The low rate of use of family planning commodities by youths is also reported in previous studies [51–53]. Nigeria, is among the countries globally with highest rates of adolescent fertility at 109 births per 1000 girls aged 15–19 per year, and yet also has one of the lowest rates of use of modern contraception in adolescents [54]. In Nigeria, 98.8% of married adolescent girls and 50.3% of unmarried sexually active adolescent girls do not use a modern contraceptive method [55]. Among the reported barriers to adolescent contraceptive use are lack of knowledge of services, cost, shyness and community stigma about sexual activity and disapproving attitudes from providers [56–58]. In Nigeria, adolescent sexual and reproductive health is affected by cultural, religious, legal, political and economic contexts [59].

A number of Nigeria health policies and other initiatives aimed at reducing disease burden in the country incorporate PPMVs as primary health care service providers [60]. As shown in this study, the PPMVs sell both antibiotics and contraceptives they are legally unauthorized to sell. Given their geographic spread, market share, and accessibility, PPMVs represent an important and often the only opportunity for access to primary health care services in most rural communities in Nigeria [7]. Consequently, despite regulations, it is common for PPMVs in Nigeria to provide services they are legally prohibited from offering, for which they have not received any training, including injectable contraceptives and dispensing antibiotics [7]. One of the main factors responsible for this is the high client patronage for these types of medicines, of which the PPMVs may be the only available source. Available report indicated that 11% of women who use injectable contraceptives noted getting their injectables from PPMVs [51]. While the majority of PPMV shops stock family planning commodities, few have received training on family planning methods, and therefore provide inadequate or often misleading counsel to their customers on family planning options or usage, and are less likely than other family planning providers to inform customers about method options or side effects [10, 51–53].

It has been argued that given the growing importance of family planning in Nigeria, contraceptives, including injectable contraceptives, should be added to the list of drugs that PPMVs are expected to stock in their shops [51]. There are calls for studies with training components for PMPVs on how to counsel clients about injectables, screen clients for eligibility, sell the method and administer injections with close supervision and monitoring by the NAPPMED and the FMOH [51–53]. Incorporating PPMVs into the health system may lead to increased use of family planning services among the target population of rural adolescents and the role of this towards UHC. This would require policy change and collaboration at the national level.

## The limitation of study

The use of a quantitative cross-sectional technique in this study was a main limitation. This is because the technique does not adequately provide context of study situation as noted in a study in Nigeria which used this technique [22]. The use of descriptive study method (qualitative or causal relationship based on a longitudinal technique) is recommended in future studies. Another limitation to this study was the use of self-assessment technique. This technique has been reported to possess some merits especially in this type of study, but its main weakness lies in the fact that it is very difficult for a respondent to be able to critically recognize and understand his or her own gap in skills and knowledge [61]. Self-assessments have also been described as subject to self-esteem bias, may be unreliable, and are difficult to validate [62].

## Conclusion and policy recommendations

The PPMVs have a large population, are in close proximity to the rural and underserved areas of Nigeria and have been included in the integrated community case management (iCCM) team. There is need to enhance their capacity through training in the proper dosage and duration of antibiotics as well as drug advice and information on proper treatment of childhood cough with antibiotics. It is also essential for the revisitation of existing policy for a possible review to expand the scope of the services the PPMVs can render. Most antibiotics used by nursing mothers to treat their children’s cough and cold were obtained from patent drug shops. These drugs also provided the treatment relief needed. More studies are encouraged in this wise to enable a change in policy regarding the types of antibiotics allowed the PPMVs to handle as well as improved monitoring and supervision channels.

This study has provided context-specific evidence on the access to medicines including family planning commodities provided by PPMVs among young people. It is obvious that there is no complete adherence to the National Drug Policy guidelines/regulatory framework on family planning services and antibiotic dispensing by the PPMVs, largely because they are the only source of these medicines in the rural areas. The study therefore makes a case for the possibility of the introduction of a training mechanism for the PPMVs that will equip them to legally dispense contraceptives and antibiotics. The study advocates for a possible policy change since these regulations may be constituting barriers to good practice. More studies are needed to determine the safety and feasibility of this practice.
